# Moonlight functions of glycolytic enzymes in cancer

**DOI:** 10.3389/fmolb.2023.1076138

**Published:** 2023-06-28

**Authors:** Petr V. Shegay, Olga P. Shatova, Anastasia A. Zabolotneva, Aleksandr V. Shestopalov, Andrei D. Kaprin

**Affiliations:** ^1^ Federal State Budget Institution, National Medical Research Radiology Center of the Ministry of Healthcare of the Russian Federation, Moscow, Russia; ^2^ Department of Biochemistry and Molecular Biology, Faculty of Medicine, Pirogov Russian National Research Medical University, Moscow, Russia; ^3^ Biochemistry Department, Peoples’ Friendship University of Russia, Moscow, Russia; ^4^ National Medical Research Centre for Endocrinology, Laboratory of Biochemistry of Signaling Pathways, Moscow, Russia; ^5^ Dmitry Rogachev National Medical Research Center of Pediatric Hematology, Oncology and Immunology, Ministry of Health of the Russian Federation, Moscow, Russia

**Keywords:** glycolytic enzymes, moonlight proteins, metabolic reprogramming, cancer signaling pathways, molecular evolution

## Abstract

Since an extensive genome research has started, basic principle “one gene—one protein—one function” was significantly revised. Many proteins with more than one function were identified and characterized as “moonlighting” proteins, which activity depend not only on structural peculiarities but also on compartmentation and metabolic environment. It turned out that “housekeeping” glycolytic enzymes show important moonlight functions such as control of development, proliferation, apoptosis, migration, regulation of transcription and cell signaling. Glycolytic enzymes emerged very early in evolution and because of the limited content of genomes, they could be used as ancient regulators for intercellular and intracellular communication. The multifunctionality of the constitutively expressed enzymes began to serve cancer cell survival and growth. In the present review we discuss some moonlight functions of glycolytic enzymes that important for malignant transformation and tumor growth.

## 1 Introduction

**FIGURE 1:**
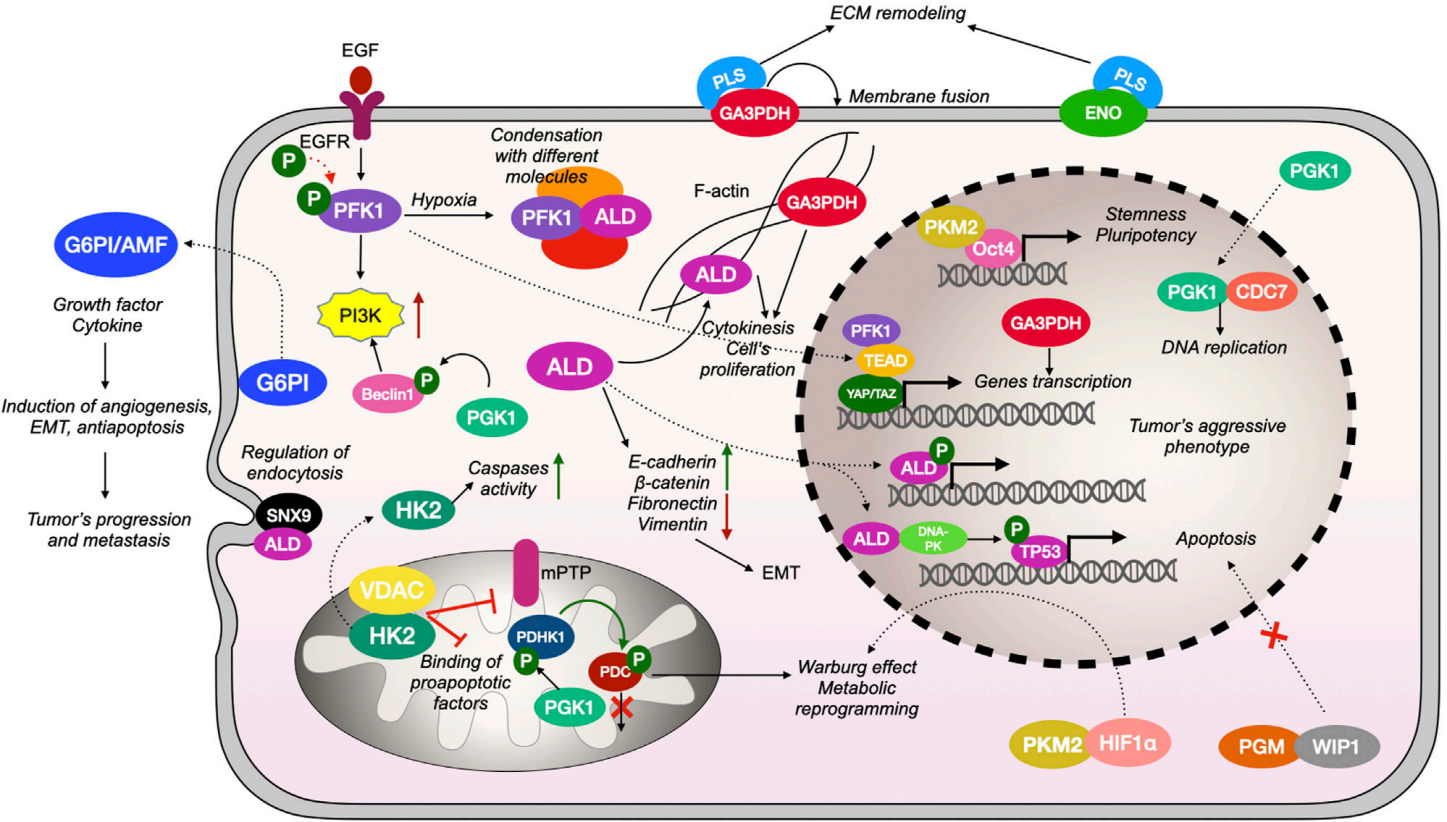
Compartmentalization and several moonlight functions of glycolytic enzymes. Mitochondrial HK2 is associated with VDAC thereby preventing the binding of pro-apoptotic factors and promoting an opening of mPTP; HK2 may also exit the cytosol where it increases caspase activity. G6PI acts as a cytokine (referred as G6PI/AMF) and growth factor outside the cell promoting angiogenesis, EMT, and preventing apoptosis. Under hypoxic conditions PFK1 forms molecular condensates, including associates with aldolase (ALD); PFK1 can be phosphorylated after EGFR activation and stimulates PI3K signaling pathway; PFK1 can be transferred into the nucleus, where it associates with transcription cofactors and influences genes expression, thus supporting tumor’s progression. ALD indirectly increases the expression of E-cadherin and β-catenin and decreases the expression of fibronectin and vimentin, thus promoting EMT; ALD binds to F-actin, which influence cytokinesis and proliferation of cancer cells; phosphorylated ALD can be transported into the nucleus where it binds to DNA, stabilizing it or promoting the expression of genes involved in tumor progression and maintaining its aggressive phenotype; ALD can also bind DNA-PK, which phosphorylates and activates TP53, thus leading to the transcription of pro-apoptotic genes. Membrane-bounded GA3PDH serves as plasminogen (PLS) receptor; nuclear GA3PDH indirectly regulates transcription of genes involved in tumor’s progression; cytosolic GA3PDH influences microtubules formation. Mitochondrial PGK1 directly phosphorylates PDHK1, which inactivates PDC thus inhibiting pyruvate utilization in mitochondria and promoting lactate production by cancer cell; cytosolic PGK1 phosphorylates beclin-1, which promotes activation of PI3K signaling pathway. Cytosolic PGM binds to phosphatase WIP1, thus preventing its translocation into the nucleus and activation of the apoptosis program. Enolase (ENO) serves as a PLS receptor and is involved in ECM remodeling and induction of angiogenesis and tumor growth. Nuclear PKM2 binds to Oct4 thereby inducing transcription of genes involved in maintaining stemness properties of the cell; cytosolic PKM2 can bind HIF1αand thus influence genes expression required for metabolic reprogramming of tumor’s microenvironment.

Glycolysis is a universal and most ancient metabolic process (Ralser, 2018). Various studies have demonstrated overexpression [2] and hyperactivation of glycolytic enzymes [3] in cancer. Importantly, the main source of energy for rapidly growing tissues as well as for stem cells indeed is the glycolytic oxidation of glucose. Furthermore, in the process of evolution, multicellular organisms began to use glycolytic enzymes not only as catalysts for the corresponding reactions, but also as instruments for a moonlighting (Kim and Dang, 2005). The multifunctioning of glycolytic enzymes has been established to be highly dependent on their compartmentalisation (Figure 1) (Jang et al., 2021). Furthermore, glycolytic enzymes, as well as glycolysis products/substrates, serve as signaling molecules (Shatova et al., 2009; Mu et al., 2018) and therefore play an important regulatory role in cell life (Kim and Dang, 2005; Jang et al., 2021).

Undoubtedly, the intensification of glycolysis is the most characteristic feature of metabolic reprogramming that cells undergo during malignant transformation (Yang et al., 2019). It has also been found that the more cancer stem cells (CSCs) in the tumor tissue, the higher the expression of glycolytic enzymes (Sancho et al., 2016), which emphasizes the role of glycolysis in ensuring the stemness of CSCs. At present, mainly it remains unexplored how compartmentalization and, accordingly, the pleiotropic properties of glycolytic enzymes are regulated, as well as whether there are any allosteric effectors of “turning on” and “turning off” the moonlight function of glycolytic enzymes. It also remains unknown when during evolution glycolytic enzymes have acquired additional functions.

The aim of our work was to summarise the evolutionary acquisition of moonlight functions by glycolytic enzymes, which are not related to the catalysis of the corresponding reactions of glycolysis.

## 2 Moonlight functioning of glycolytic enzymes

### 2.1 Hexokinase (HK) is involved in apoptosis

The first enzyme of glycolytic conversion of glucose, hexokinase (HK), has mitochondrial localization in malignant tumors in more than 50% of cases, as it was shown for the HK2 isoenzyme. The mitochondrial form of this enzyme is involved in the implementation of the apoptosis programme (Bustamante and Pedersen, 1977). HK2 is expressed mainly in embryonic tissues, as well as in adipose and muscle tissues, and is almost not present in other tissues. It is important to note that this isoenzyme is overexpressed in tumors of various localizations (Wang et al., 2017). Furthermore, suppression of HK2 expression was found to lead to a decrease in tumour cell proliferation in xenograft models (Wang et al., 2017).

Inhibition of protein kinase B (PKB or Akt) phosphorylation in photoreceptors has also been shown to lead to the movement of mitochondrial HK2 into the cytoplasm, increased caspase activity, and decreased cell viability. Photoreceptors are characterized by the presence of the Warburg effect, unlike other terminally differentiated neurons. HK2, muscle pyruvate kinase 2 (PKM2), and LDH-A are three enzymes responsible for the realization of this effect (aerobic glycolysis) (Weh et al., 2020). Therefore, HK2 is normally found in mitochondria and is associated with a voltage-gated anion channel (VDAC), where it gains preferential access to ATP for glucose phosphorylation. Being associated with VDAC, HK2 can also prevent the binding of pro-apoptotic factors to mitochondria and the opening of the mitochondrial permeability transition pore (mPTP), which blocks the release of cytochrome c (Majewski et al., 2004). Akt has been shown to modulate the ability of HK2 to bind to VDAC through its direct phosphorylation (Rajala et al., 2013). It has been established that in photoreceptor rod cells lacking HK2, activation of HK1 occurs (Weh et al., 2020). HK2-deficient photoreceptors are more susceptible to acute nutritional deficiency in an experimental model of retinal detachment, and HK2 is important to maintain photoreceptor functions during aging (Majewski et al., 2004; Weh et al., 2020).

### 2.2 Glucose-6-phosphate isomerase (G6PI) regulates cell motility

The second glycolytic enzyme, which converts glucose-6-phosphate to fructose-6-phosphate, phosphoglucoisomerase (glucose-6-phosphate isomerase, G6PI), also performs some additional important functions. This protein is known as autocrine motility factor (AMF), neuroleukin, neurotrophic growth factor, maturity factor that determines the differentiation of human myeloid cells and the ability of tumor cells to metastasize (Masumura et al., 1982; Osthus et al., 2000; Yanagawa et al., 2004a; Sato et al., 2008). In cancer patients, the activity of this glycolytic enzyme is significantly increased in the blood, making it possible to use it as an early marker in cancer diagnostics. G6PI also possesses antiapoptotic properties (Chiao et al., 1999), which are extremely important for tumor cell survival. The high activity of G6PI ensures the survival, but not proliferation, of embryonic spinal and sensory neurones (Yanagawa et al., 2004a). G6PI also serves as a maturation factor: it stimulates monocyte maturation, induces the secretion of immunoglobulins, affects osteoblast differentiation, and causes arthritis in mice. It draws attention to the fact that a specific inhibitor of G6PI enzymatic activity, mannose-6-phosphate, also inhibits the cytokine functions of this enzyme, namely the regulation of cell motility, anti-apoptosis, proliferation, etc. (Yanagawa et al., 2004b).

G6PI acts as a cytokine and growth factor in a wide variety of extracellular processes (Zong et al., 2015). AMF/G6PI is a multifunctional cytokine that exhibits multifunctional growth factor-like activity through binding with a unique cognate seven-transmembrane 78 kDa glycoprotein receptor (glycoprotein 78, gp78) (autocrine mobility factor receptor, AMFR) (Shimizu et al., 1999). Many studies have shown that AMF not only stimulates the motility of AMF-producing tumour cells in an autocrine manner but also acts as a paracrine factor in venous endothelial cells. Thus, AMF induces angiogenesis by stimulating cell motility and increases the expression of vascular endothelial growth factor receptors (VEGFR) (Funasaka et al., 2001). Overexpression of AMF/G6PI and AMFR has been found in a wide range of malignancies and is associated with tumour progression, metastasis, and angiogenesis (Kaynak et al., 2005).

### 2.3 Phosphofructokinase-1 (PFK-1) regulates transcription factors activity

Phosphofructokinase 1 (PFK-1) catalyzes the third reaction of glycolysis and is the second regulatory enzyme after the hexokinase reaction. PFK-1 limits the rate of glucose oxidation and simultaneously performs an antioxidant function (Mazzio and Soliman, 2003). There are many indications of increased expression of PFK-1, as well as its regulatory protein PFK-2, in different types of cancers, such as lung cancer (Shen et al., 2020), breast cancer (Onodera et al., 2014), hepatocellular carcinoma (Feng et al., 2020). One of the most important moonlight functions of PFK-1 is the mediation of phosphatidylinositol-3-kinase (PI3K) activation. The platelet isoform of PFK-1 (PFKP) was shown to be phosphorylated after activation of the epithelial growth factor receptor (EGFR), leading to binding of PFKP to the N-terminal SH2 domain of p85α and therefore activation of PI3K (Lee et al., 2018). Importantly, high expression of the PFKP isoform is often observed in lung cancer tissues and cell lines and is associated with a poor prognosis (Shen et al., 2020).

Furthermore, PFK-1 has been shown to bind to the TEADs transcriptional cofactors, which promotes their cooperation with YAP/TAZ transcription factors in human breast cancer cell lines. YAP/TAZ are key transcription factors that regulate cancer cell proliferation and tumours aggressiveness; therefore, their activation results in gene transcription that maintains tumour progression (Shen et al., 2020).

It should be noted that in the cells of multicellular organisms, glycolytic proteins are not just diffusely distributed in the cytosol; on the contrary, there are regulatory mechanisms that organise glycolytic proteins *in vivo*. The proposed organization of glycolytic proteins *in vivo* may have important implications for the subcellular regulation of this metabolic pathway and its enzymatic activity (Zecchin et al., 2015). PFK-1 is diffusely localized in the cytosol of cells. However, during hypoxia and, as a result, energy stress, the enzyme can move dynamically with the formation of biomolecular condensates. When returning to normoxic conditions, PFK-1 is dispersed again in the cytosol. It has been established that PFK-1 condensates have liquid properties and that their molecular dynamics, including viscosity and biophysical properties, change during prolonged hypoxic conditions. It was also found that PFK-1 and aldolase-1 interact by recruiting one each other into condensates, which indicates necessity of self-association event that triggers a feed-forward loop for the recruitment of glycolytic proteins into condensates (Zecchin et al., 2015; Pirovich et al., 2021).

### 2.4 Aldolase regulates the epithelial-mesenchymal transition

Aldolase is known to exist in three isoforms expressed in a tissue-dependent manner: aldolase A (ALDOA) is expressed mainly in muscles; aldolase B is expressed in the liver; aldolase C is expressed in nervous tissue. ALDOA is known to be the most abundant aldolase isoform in almost all cancers (Chang et al., 2018). In addition to its function in glycolysis, ALDOA possesses many moonlight functions, such as signal transduction (Kim et al., 2002), vesicle trafficking (Kao et al., 1999), and cell motility (Buscaglia et al., 2006). For example, in some mammalian cells, aldolase can contribute to the formation of the intracellular cytoskeleton, for example by interacting with F-actin and vacuolar-type H^+^-ATPase (V-ATPase) or by inhibiting Wiskott-Aldrich syndrome protein (WASP)-dependent actin polymerisation (Pirovich et al., 2021) leading to a cytokinesis defect (Lew and Tolan, 2012). Aldolase can also influence epithelial-mesenchymal transition (EMT) by decreasing expression of E-cadherin and β-catenin, and by simultaneously increasing fibronectin and vimentin formation, that promotes carcinogenesis (Du et al., 2014).

In many types of tumours, ALDOA is located in the nucleus (Satou et al., 2004). The mechanism of such compartmentation of ALDOA is not known for sure, but it was shown ALDOA is associated with AT-rich DNA sequences in nucleus (Satou et al., 2004), that may point to its role in regulation of transcription and cell proliferation (Gizak et al., 2019).

Aldolase is also required for endocytosis regulation by binding to the intracellular transport protein nexin 9 (SNX9) (Rangarajan et al., 2010). Aldolase has been reported to interact with phospholipase D2, heparin, and γ-tubulin (Pirovich et al., 2021), but the functional role of these interactions is still unknown.

Aldolase may also affect the activities of AMP-activated protein kinase (AMPK) (Zhang et al., 2017) and fructose-bisphosphatase 2 (FBP2) (Gizak et al., 2008) which makes aldolase an effective sensor of glucose availability. It was found that at a low intracellular concentration of fructose-1,6-bisphosphate, a substrate of aldolase, this glycolytic enzyme interacts with V-ATPase. This interaction in turn promotes the activation of AMPK, which consequently stimulates ATP production by increasing the activity or expression of proteins involved in catabolic pathways (M. Li et al., 2019).

In tumor cells, aldolase can promote the activation of DNA-dependent protein kinase (DNA-PK) by binding to its catalytic subunit. In turn, DNA-PK phosphorylates the tumor suppressor protein p53, increasing its activity (Ma et al., 2018). Aldolase can also activate the Wingless/Int-1 (Wnt) oncogenic signaling pathway in mammalian cells by suppressing the elimination of the β-catenin signaling molecule involved in the development of colorectal cancer (Caspi et al., 2014).

Furthermore, ALDOA can (J. Li et al., 2019) act as an oncogene in bladder cancer by interacting with E-cadherin-epidermal growth factor (EGFR) signaling, and this event leads to metastasis. Thus, aldolase has both pro-oncogenic (through Wnt/E-cadherin-EGFR signaling) and anti-oncogenic (through p53 activation) functions in different types of cancers.

### 2.5 Triose phosphate isomerase (TPI) regulates the distribution of metabolites

TPI is hyperexpressed in some cancers, such as lung cancer (Chen et al., 2002), squamous cell lung carcinoma (Li et al., 2006), urinary cancer (Unwin et al., 2003), and chemoresistant ovarian carcinoma (di Michele et al., 2010). The functioning of TPIs is essential for the regulation of the distribution of metabolites between glycolysis and pentose phosphate pathway (PPP). Activation of TPI leads to ATP production through glycolysis, whereas its inhibition directs dihydroxyacetone phosphate toward the PPP (Grüning et al., 2014), the main pathway of reduced NADPH formation (Ying, 2008), which is important for many anabolic processes, as well as essential for antioxidant defence and maintenance of redox potential (Ying, 2008). Furthermore, PPP is a key source of ribose for the gene transcription during stress conditions (Krüger et al., 2011). Importantly, TPI was proposed to be used as an antidrug-resistant agent in gastric cancer and a target candidate for gastric cancer treatment (Wang et al., 2008).

### 2.6 Glyceraldehyde-3-phosphate dehydrogenase (GA3PDH) is involved in membrane transport and plasminogen binding

Glyceraldehyde-3-phosphate dehydrogenase (GA3PDH) catalyzes the oxidation of glyceraldehyde-3-phosphate, the fifth reaction of glycolysis. This enzyme exhibits many different activities not related to glycolysis. For example, GA3PDH has been shown to have uracil DNA glycosylase activity (Wang et al., 1999; Giegé et al., 2003). Furthermore, the function of the protein depends on its localization (the protein can be dissolved either in the cytosol or nucleus, or anchored in the membrane) (Mazzola and Sirover, 2002). GA3PDH is involved in membrane fusion, formation of microtubules, construction of telomeres (Sirover, 2005); it possesses kinase activity, exports RNA from cell nuclei, participates in replication and repair, apoptosis, development of age-dependent neurodegenerative diseases and, ultimately, in malignant transformation of cells (Sirover, 1999, 2005). It should be noted that this enzyme is one of the most sensitive targets for nitric oxide (NO) (Mazzola and Sirover, 2002; Sirover, 2005). Each of the intracellular processes mentioned above requires the inclusion of GA3PDH in a series of multienzyme complexes, while the enzyme retains its regular structure. Importantly, GA3PDH interacts stoichiometrically and specifically with the muscle and cardiac isoenzymes of LDH. The complex of these enzymes becomes poorly soluble and can affect the balance of NAD^+^/NADH and the entire glycolytic pathway of glucose oxidation (Svedružić and Spivey, 2006).

GA3PDH was shown to be an evolutionary conserved plasminogen receptor in mammalian cells (Chauhan et al., 2017). Due to the ability of extracellular bacterial GA3PDH to bind plasminogen, plasmin (the active form of plasminogen) can degrade the extracellular matrix (ECM), thus promoting bacterial invasion and migration. Therefore, GA3PDH is an important factor of pathogenicity and virulence (Franco-Serrano et al., 2021). Increased expression of GA3PDH is also associated with tumour aggressiveness in some cancers (Heiden et al., 2009). A similar mechanism engaging plasminogen binding by GA3PDH followed by ECM remodeling may be used by cancer cells and tumor-associated macrophages to promote metastasis and cancer progression (Chauhan et al., 2017).

### 2.7 Phosphoglycerate kinase possesses protein kinase activity and regulates transcription

Phosphoglycerate kinase 1 (PGK1) in glycolysis catalyzes the conversion of 1,3- bisphosphoglycerate to 3-phosphoglycerate with generating of 1 mole of ATP. At the same time PGK1 possesses many features of oncogene and promotes tumor growth, as well as cancer cells migration and invasion (Fu and Yu, 2020). PGK1 expression may serve as a prognostic marker of tumour progression considering the strong correlation of its high expression in many tumours, including breast cancer, colon cancer, glioma, lung cancer, and liver cancer, with tumour proliferation, metastasis, occurrence, development, and poor prognosis (Tang et al., 2008; Ahmad et al., 2013; Shashni et al., 2013; Ding et al., 2014). Furthermore, it was proposed to use PGK1 as a therapeutic target for tumours (Gou et al., 2021).

PGK1 not only provides energy required for tumor growth, but also act as a protein kinase. Mitochondrial PGK1 directly phosphorylates pyruvate dehydrogenase kinase isoenzyme 1 (PDHK1), which in turn phosphorylates and inactivates the pyruvate dehydrogenase complex (PDC). Thus, the conversion of pyruvate to acetyl-CoA in the mitochondria is inhibited, that increases lactate production and promotes the Warburg effect in tumor cells (Wilson et al., 2019). Additionally, PGK1 phosphorylates Beclin-1, which activates PI3K signaling pathway, conducing the tumor progression (Qian et al., 2017).

PGK1 is also a regulator of transcription and replication. For instance, PGK1 is the upstream regulator of β-catenin, which is known to be a tumor-associated oncoprotein that affects tumor growth, invasion, metastasis, angiogenesis (Lowy et al., 2006; Lincet and Icard, 2015). Besides, PGK1, being transferred into the nucleus, can bind the kinase cell division cycle 7 (CDC7) protein and convert ADP to ATP. Therefore, it stops the inhibition of CDC7-ASK activity by ADP, that allows DNA helicase to be recruited to the starting point of replication, hence DNA replication and cell proliferation of tumor cells become possible (Li et al., 2018).

### 2.8 Phosphoglycerate mutase (PGM) is involved in the regulation of antiapoptosis

It has been established that phosphoglycerate mutase-1 (PGM-1) is overexpressed in gliomas, where it increases the efficiency of the DNA damage response pathway (DDR) due to cytoplasmic binding with phosphatase WIP1 (protein phosphatase 2C delta), thereby preventing its nuclear translocation and subsequent dephosphorylation of the proteins of ATM signaling pathway. Suppression of FGM-1 expression in glioma cells reduces the formation of foci of γ-H2AX (phosphorylated form of H2A), increases apoptosis, and reduces clonogenicity after irradiation and treatment with temozolomide (Ohba et al., 2020).

### 2.9 Enolase is a plasminogen receptor

α-Enolase (ENO1) is one of the most important enzymes in the glycolytic pathway, and at the same it is a multifunctional oncoprotein contributing to hit seven “hallmarks of cancer” (Huang et al., 2022) Moonlighting of ENO1 is involved in deregulating of cellular energetic, sustaining of tumor proliferation, inhibiting of cancer cell apoptosis, as well as evading of growth suppressors and immune surveillance. Besides, ENO1 is expressed on the surface of several cell types, where it acts as a plasminogen receptor, concentrating plasmin proteolytic activity on the cell surface (Perconti et al., 2017), and thus ENO1 is involved in extracellular matrix (ECM) remodeling, cancer invasion, and metastasis by inducing angiogenesis (Huang et al., 2022).

Functions of ENO1 are strongly dependent on its localization. Membrane-anchored ENO1 acts as a plasminogen receptor which converts plasminogen into plasmin and promotes ECM degradation, cancer cells invasion, migration and metastasis (Hsiao et al., 2013). At the centrosome, ENO1 is essential for cytoskeleton organization (Georges et al., 2011). Cytosolic ENO1 in addition to participating in glycolysis, is also necessary for maintaining mitochondrial membrane stability (Didiasova et al., 2019) and regulation of intracellular signaling pathways (Díaz-Ramos et al., 2012). ENO1 can be secreted in the extracellular space, where it associates with exosomes (Didiasova et al., 2015).

Alternative translation of *ENO1* gene produces Myc-binding protein 1 (MBP1), which is not involved in glycolysis but suppresses the expression of *c-Myc* proto-oncogene (Subramanian and Miller, 2000). Notably, that exposure to epidermal growth factor (EGF) and lipopolysaccharide (LPS) has been shown to promote α-enolase expression (Perconti et al., 2017).

### 2.10 Pyruvate kinase (PK) is responsible for proliferation

Pyruvate kinase (PK) catalyzes the tenth reaction of glycolysis and irreversibly converts phosphoenolpyruvate to pyruvate; this is the third regulatory reaction of the process. In addition to its enzymatic function, this protein localizing inside the nucleus regulates the proliferation process (Hoshino et al., 2007). M2 isoform of PK (PKM2) is expressed in the cells and tissues with high proliferating rate, including embryonic and tumor cells (Mazurek et al., 2005). It was shown that PKM2 may exist in dimeric and tetrameric forms: dimeric PKM2 emerges protein kinase activity (with phosphoenolpyruvate as phosphate donor), while PKM2 tetramer is a PK. Indeed, dimeric isoform’s expression promotes cellular proliferation indicating that its protein kinase activity is essential for cells division (Mazurek et al., 2005). During tumorigenesis PKM2 acts as an important factor for maintaining stemness phenotype of cancerous cells due to its ability to interact with octamer-binding transcription factor 4 (Oct-4) and enhance its transactivation (Lee et al., 2008). The Oct-4 protein is known to be an important factor in maintaining pluripotency in embryonic stem cells. Furthermore, in different works, it was shown that nuclear PKM2 interacts with hypoxia-inducible factor-1α (HIF-1α), thus increasing the expression of its target genes (such as GLUT1 and LDHA) and providing metabolic reprogramming of tumour cells (Luo et al., 2011; Wang et al., 2014).

PKM2 is also involved in histone modifications to control the expression of c-Myc and cyclin D1 in malignant cells upon EGF signaling (Yang et al., 2012).

In addition to protein kinase activity, PKM2 is also involved in the production of cyclic adenosine monophosphate (cAMP) due to the activation of soluble adenylyl cyclase (sAC). Increasing the cAMP level results in upregulation of β_1_-integrin pathway and loss of cell polarity (Onodera et al., 2014). Therefore, different activities of PKM2 orchestrate the malignant transformation pathway necessary for tumour development.

### 2.11 Lactate dehydrogenase (LDH) provides stemness and inhibits apoptosis

In the literature, there are indirect indications of a moonlight functions of LDH, the last key enzyme of glycolysis. Numerous studies show that LDH-A (an enzyme isoform that predominantly catalyzes the conversion of pyruvate to lactate) has an aberrantly high level of expression in many cancers and is often used as a marker of an unfavourable prognosis of the disease (Urbańska and Orzechowski, 2019). There are several explanations for the molecular mechanisms of action of LDH-A, which ensure the division and survival of tumor cells. First, LDH-A function is needed to provide malignant cells with sufficient energy. It is well known that tumour cells significantly increase the rate of glycolysis and lactate formation under hypoxic and normoxic conditions (the Warburg effect) (Brooks, 2018). Under normoxic conditions, the cell proliferation rate decreased after suppression of LDH-A, and under hypoxia conditions (0.5% oxygen), tumor cell growth with LDH-A deficiency was also severely impaired; tumor cells with reduced LDH-A activity could not maintain high levels of ATP, which probably contributed to the slowing of cell proliferation under normoxic or hypoxic conditions. Secondly, LDH-A is involved in maintaining the stem properties of tumor cells. The expression of LDH-A is largely associated with Oct-4, which plays an important role in the self-renewal of embryonic stem cells. At the same time, LDH-A knockdown can reduce Oct-4 expression and oncogenic properties of cells *in vitro* and *in vivo* (Zhang et al., 2012).

In addition, LDH-A indirectly promotes tumor survival by protecting it from ROS damage, and LDH-A can directly inhibit apoptosis. An immunohistochemical study of melanoma by Zhuang et al. (2010) showed that the expression of LDH-A strongly correlates with the expression of the antiapoptotic proteins Mcl-1 and Bcl-XL, while the elimination of LDH-A increases the cleavage of poly-(ADP-ribose) polymerase (PARP) and reduces the expression of the X-linked inhibitor of apoptosis protein (XIAP), Bcl-2 and Bcl-XL, which led to a decrease in the oncogenicity of the pancreatic cell line BxPC-3 (Rong et al., 2013). In xenografts of breast cancer cell lines, LDH-A knockout was also found to increase levels of Bcl-2-associated protein X (Bax), PARP, caspase-9, cytoplasmic cytochrome C, and superoxide anion, while Bcl-2 expression and mitochondrial membrane potential were reduced (Wang et al., 2012). Finally, LDH-A overexpression can also promote tumour growth by preventing necrosis under hypoxic conditions. Lewis et al. (2000) indicated that tumours overexpressing LDH-A and Rcl protein have a small area of necrosis compared to tumours overexpressing Rcl and VEGF. This indicates that increased LDH-A expression protects central tumor cells from hypoxia-induced necrosis. LDH-A significantly influences malignant cell invasion and migration through regulation of key participants in these cellular processes, for example, by causing ECM degradation by stimulating metalloproteinase-2 (MMP-2) production, promoting metastatic vasculogenesis through activation of VEGF, and inhibiting cell adhesion through suppression of E-cadherin expression. In addition, EMT activation also underlies the contribution of LDH-A to cancer metastasis (Jiang et al., 2016). Jiang et al. found that LDH-A knockdown prevents tumor cell invasion, which is accompanied by a decrease in Snail, N-cadherin, fibronectin, and vimentin expression, but an increase in E-cadherin expression in bladder cancer (Jiang et al., 2016). Several studies have shown that LDH-A can also regulate tumour angiogenesis. The regulation of angiogenesis by LDH-A is mainly dependent on the production of lactic acid. Acidification of the microenvironment promotes the production of interleukin-8 (IL-8) and VEGF, and uptake of lactic acid by vascular endothelial cells triggers IκBα phosphorylation/degradation, activates nuclear factor-kappa B (NF-κB), promotes IL-8 expression, and subsequently accelerates angiogenesis and tumor growth (Shi et al., 2001). In our opinion, the results described above indicate that tumour progression is provided so much by creating an acidic environment, as well as by the presence of functionally active LDH in the cell (moonlighting of the enzyme) or is provided by a high intracellular level of lactate, the product of LDH.

## 3 Conclusion

**FIGURE 2:**
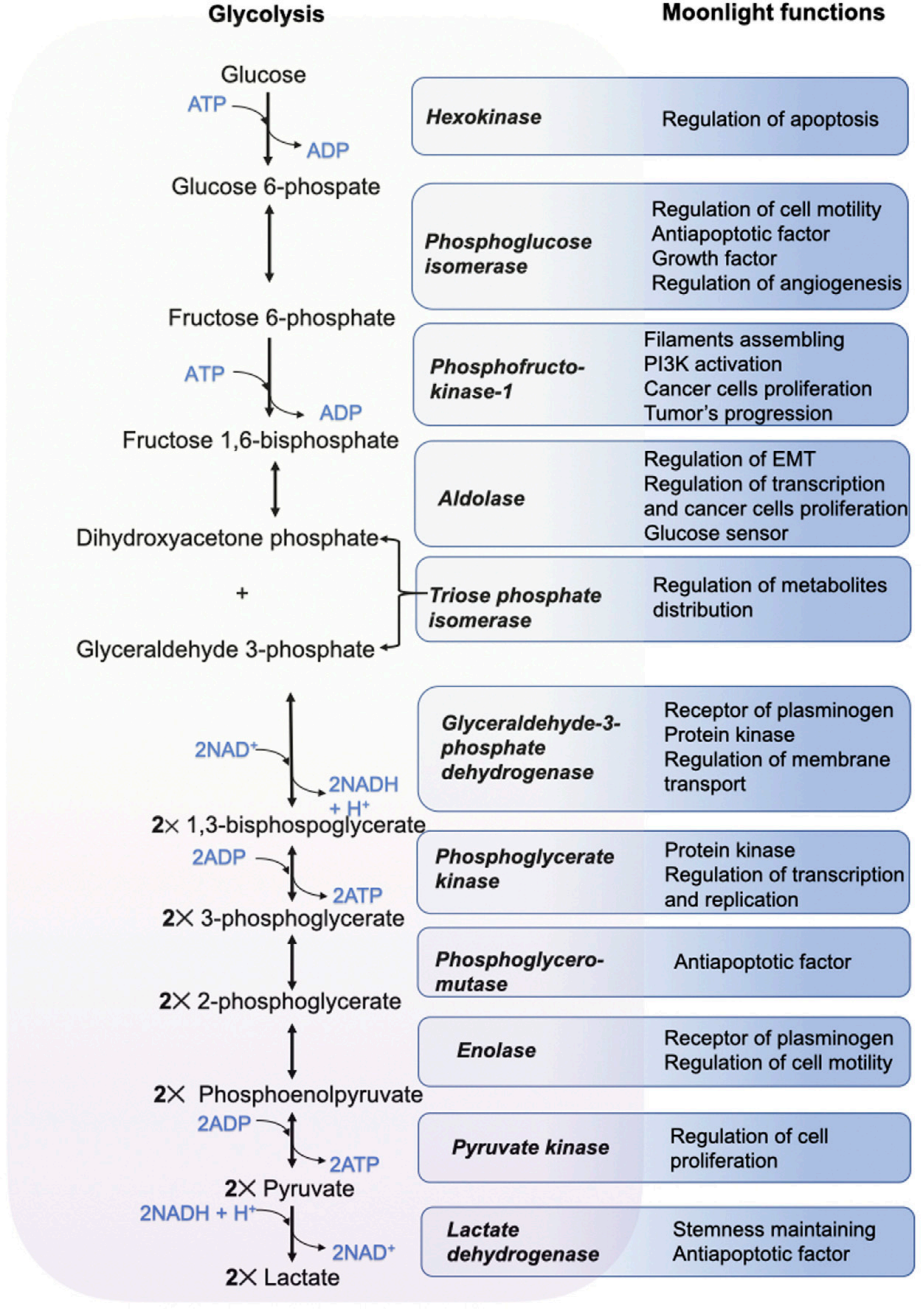
Some moonlight functions of glycolytic enzymes important for malignant transformation and tumor progression.

Since extensive investigations of procaryotic and eucaryotic genomes have started, the traditional idea of “one gene—one protein—one function” has lost its original meaning. Glycolytic enzymes as well as many other proteins belong to moonlighting enzymes whose physiological role is not limited to catalytic function, but which can serve as regulators of a variety of cellular processes (Figure 2).

The change in moonlight activity of the enzyme depends on many stimuli such as a change in temperature, a change in the redox state of the cell, a change in the oligomeric state of the protein, direct interactions with a variety of binding partner proteins, or changes in the cellular concentration of a ligand, substrate, cofactor, or product (Jeffery, 2009). Besides, functions of some glycolytic proteins can be regulated through non-coding RNAs activity (Liu et al., 2018) or due to post-translational modifications (Zhan et al., 2015; Zakrzewicz et al., 2018). The subcellular localization of moonlighting proteins or its export in the extracellular matrix is also crucial for determining its catalytic or regulatory activity.

The fact that glycolytic enzymes’ moonlight activity is pronounced in cancer cells, points at their exclusive role in malignant transformation. Although, the first enzyme of glycolysis, hexokinase, demonstrates lower expression and activity compared to other glycolytic enzymes (Patra et al., 2013). Thus, HK controls the flux through and limits the rate of glycolysis. Hence, the overexpression of glycolytic enzymes observed in tumours cannot speed up glucose catabolism but is likely associated with other non-catalytic processes.

Overexpression of almost all glycolytic enzymes in cancer cells contribute to most of the hallmarks of cancer (Hanahan, 2022), such as sustaining proliferative signaling, evading growth suppressors, avoiding immune destruction, enabling replicative immortality, tumour-promoting inflammation, activating invasion and metastasis, inducing or accessing vasculature, resisting cell death, deregulating cellular metabolism (Heiden et al., 2009; Bu et al., 2018; Ciscato et al., 2021; Huang et al., 2022).

We consider that cancerous cells reprogram their microenvironment such a way to induce the switching of functional activity of glycolytic enzymes and direct their action to provide tumor progression. For instance, the muscle isoform ALDOA is one of the most abundant glycolytic enzymes in almost all cancer cells (Chang et al., 2018), and participates in many cellular processes providing cancer cell survival, proliferation, tumour invasion, and metastasis. Furthermore, inhibition of ALDOA activity by using a pharmacological inhibitor or silencing leads to the death of cancer cells due to disruption of the integrity of their actin cytoskeleton, which is dependent on aldolase activity (Gizak et al., 2019). Thus, inhibition of ALDOA activity is most deleterious to cells undergoing EMT, a process that provides cancer cell invasion, and can be used as a universal approach to anticancer therapy. The similar effect was observed for colon carcinoma using a zebrafish-based system of human tumor xenograft analysis (Jung et al., 2012). The invasion and migration of cancer cells were shown to be suppressed by inhibition of the action of enolase, without producing toxic side effects.

Therefore, the Warburg effect observed in many cancers and emerging in glycolysis intensification and hyperexpression of glycolytic enzymes may have a crucial meaning for glycolytic enzymes moonlighting. Increased lactate production as well as acidification of the microenvironment and other metabolic changes that occurred during tumour growth (Shegay et al., 2022) may induce functional switching of glycolytic enzymes to stimulate their moonlighting. Due to moonlight action, the proteins will induce their own expression, promote tumour cell transformation to a stemlike phenotype, and stimulate their proliferation, invasion, migration, and resistance to therapy agents. Therefore, the investigation of protein multifunctionality is very important for selecting a target for cancer therapy.

At present many questions remain unsolved. How abundant are moonlighting proteins? What allows a protein to switch a particular function to moonlight function? How do moonlighting functions evolve? We cannot answer these questions now, but we understand that moonlighting proteins functioning form a new level of complexity and integrity in the cell. Furthermore, the high expression and exporting of some glycolytic enzymes on a cell surface or extracellular space make them very attractive diagnostic and therapeutic targets. Thus, understanding the exact functions of the enzyme and the conditions of its function switching is very important to improve the strategies of the anticancer drug search.
